# Pink1-Mediated Chondrocytic Mitophagy Contributes to Cartilage Degeneration in Osteoarthritis

**DOI:** 10.3390/jcm8111849

**Published:** 2019-11-02

**Authors:** Hyo Jung Shin, Hyewon Park, Nara Shin, Hyeok Hee Kwon, Yuhua Yin, Jeong-Ah Hwang, Hee-Jung Song, Jinhyun Kim, Dong Woon Kim, Jaewon Beom

**Affiliations:** 1 Department of Medical Science, Chungnam National University College of Medicine, Daejeon 35015, Korea; shinhyo1013@gmail.com (H.J.S.); phw6304@gmail.com (H.P.); s0870714@gmail.com (N.S.); kara00124@gmail.com (H.H.K.); yoonokhwa527@gmail.com (Y.Y.); ijjanghwang@gmail.com (J.-A.H.); 2 Department of Anatomy and Cell Biology, Brain Research Institute, Chungnam National University College of Medicine, Daejeon 35015, Korea; 3 Department of Neurology, Chungnam National University College of Medicine, Daejeon 35015, Korea; nrsono@cnu.ac.kr; 4 Division of Rheumatology, Department of Internal Medicine, Chungnam National University College of Medicine, Daejeon 35015, Korea; md228@hanmail.net; 5 Department of Physical Medicine and Rehabilitation, Chung-Ang University Hospital, Chung-Ang University College of Medicine, Seoul 06973, Korea

**Keywords:** osteoarthritis, MIA (monosodium iodoacetate), mitophagy, Pink1, mitochondrial dynamics

## Abstract

Cartilage loss is a central event in the pathogenesis of osteoarthritis (OA), though other than mechanical loading, the biochemical mechanisms underlying OA pathology remain poorly elucidated. We investigated the role of Pink1-mediated mitophagy in mitochondrial fission, a crucial process in OA pathogenesis. We used a monosodium iodoacetate (MIA)-induced rodent model of OA, which inhibits the activity of articular chondrocytes, leading to disruption of glycolytic energy metabolism and eventual cell death. The OA rat cartilage exhibits significant induction of autophagy-related proteins LC3B and p62, similar to human osteoarthritic cartilage. Moreover, expression of Pink1 and Parkin proteins were also increased in OA. Here, we confirm that Pink1-mediated mitophagy leads to cell death in chondrocytes following MIA treatment, while deficiency in Pink1 expression was associated with decreased cartilage damage and pain behaviors in MIA-induced OA. Finally, we found that autophagy and mitophagy-related genes are highly expressed in human osteoarthritic cartilage. These results indicate that OA is a degenerative condition associated with mitophagy, and suggest that targeting the Pink1 pathway may provide a therapeutic avenue for OA treatment.

## 1. Introduction

Osteoarthritis (OA) is a complex disease involving the whole synovial joint tissue. OA is the most common form of arthritis, characterized by cartilage destruction, as well as synovial inflammation and subchondral bone remodeling [1,2]. Animal models of OA have greatly improved our understanding of its pathogenesis and treatment. Among the various OA induction methods, chemically-induced OA models primarily involve the injection of toxic or inflammatory compounds directly into the knee joint to elicit intra-articular inflammation, direct matrix damage, or chondrocyte toxicity [3]. Papain, monosodium iodoacetate (MIA), quinolone, and collagenase are among the most common chemicals used to induce OA in animals. Their ease of induction is advantageous for designing reproducible studies. Among the most well-recognized methods for inducing OA is the intra-articular injection of MIA, an inhibitor of glyceraldehyde-3-phosphate dehydrogenase. MIA has been shown to induce chondrocyte death, leading to a loss of proteoglycan matrix and the formation of cartilage lesions, resulting in functional joint impairment that closely resembles conditions seen in human OA [4].

Autophagy plays an important role in maintaining cellular homeostasis via the removal of misfolded or aggregated proteins, and clearing damaged organelles, such as mitochondria, endoplasmic reticulum, and peroxisomes, as well as eliminating intracellular pathogens. While autophagy is generally regarded as a survival mechanism, its dysregulation has been linked to non-apoptotic cell death [5,6,7]. Induction of autophagy is regulated by a series of autophagy-related genes, including light chain 3 (LC3) and p62. Recent evidence suggests that the dysregulation of autophagy is closely related to the pathogenesis of OA [8,9].

Mitophagy is the selective engulfment of mitochondria by auto-phagosomes and their subsequent catabolism by lysosomes. It was initially found that mitochondria are selectively engulfed by auto-phagosomes following a loss in membrane potential [10], suggesting that mitophagic processes regulate the selective removal of damaged mitochondria. One of the initial events in mitophagy is the differentiation between damaged and normal mitochondria. Following their identification by PTEN-induced kinase 1 (Pink1), defective mitochondria are engulfed in double-membraned auto-phagosomes that fuse with lysosomes, thereby merging their contents and allowing hydrolytic degradation [11]. The presence of Pink1 on the mitochondrial surface is enhanced by mitochondrial damage and depolarization, which prevents mitochondrial import of Pink1 and its presenilins-associated rhomboid-like protein (PARL)-dependent cleavage [12]. Induction of Pink1 expression has been associated with increases in neuropathic pain, and is involved in both the induction and maintenance of neuropathic pain caused by cell death [13]. Recent studies have also identified a potential role for mitophagy in chondrocytes [14,15]. Based on these observations, we sought to investigate the role of mitophagy and Pink1 expression in cartilage damage in OA.

## 2. Materials and Methods

### 2.1. Animals and Arthritis Models

Sprague-Dawley rats (6-week-old males, 150–200 g) were obtained from Damul Science company (Daejeon, Korea) and adapted to the study habitat for 1 week prior to our experiment. Animals were group housed in a temperature-controlled room with a 12/12 h day/night cycle. MIA (Sigma-Aldrich, St Louis, USA) was injected into the intra-articular space through the infrapatellar ligament of the right knee. Animals were anesthetized with 5% isoflurane followed by 2.5% maintenance during the MIA injection procedure. MIA was dissolved in 20 µL saline and administered using a 30-gauge needle. Sham MIA-OA animals received an intra-articular injection of saline. Naïve animals were used for comparison in the immunohistochemistry experiments. Pink1-deficient mice were provided by Dr. Eun-Hye Joe [16]. Heterozygous mice on C57BL/6 mixed background were bred to generate Pink1-null mice; their wild type (WT) littermates were used as controls. All experiments were approved by the Institutional Animal Care and Use Committee of Chungnam National University. 

### 2.2. Histology and Immunohistochemistry

Cartilage from humans with OA was frozen, cut into 4-μm sections and fixed in 4% paraformaldehyde (PFA). Knee joint cartilage from MIA-injected rats and Pink1-deficient mice were fixed in 4% (v/v) PFA for 2 days, decalcified in Calci-Clear solution (catalog no. HS-105; National Diagnostics, USA) for 2 days, sectioned in the coronal plane (4 µm thickness), embedded in paraffin wax, and used to prepare slides after staining with hematoxylin. Sections were also stained with Fast Green (catalog no. 2353-45-9; Sigma-Aldrich, St. Louis, MO, USA) and Safranin-O (catalog no. CI-50240; Junsei Chemical Co. Tokyo, Japan) to evaluate the extent of histopathological lesions. For histological analysis, slides were sequentially dehydrated in 70%, 80%, 90%, and 100% ethanol. Finally, sections were cleared in xylene. A light microscope and a digital camera were used to capture and evaluate the histopathological features of the articular cartilage. 

### 2.3. Chondrocyte Isolation and Culture Condition 

Primary chondrocytes were isolated from the femoral condyles and tibial plateaus of human patients, as previously described [17]. Briefly, we used a scalpel to excise cartilage from the femoral condyles and tibial condyles of OA patients who received total knee replacement arthroplasty at Chungnam National University Hospital. The study protocol was approved by the Institutional Review Board (IRB No. 2016-06-007). Excised cartilage was cut into 2-mm thick sections and the digested suspension passed through a 40 µM pore size cell strainer to isolate individual chondrocytes. Cells were counted using a cell counter. Cells (5 × 10^6^) were seeded into 10-mm diameter dishes and cultured for 10 days in Dulbecco’s minimal essential medium supplemented with 10% (v/v) fetal bovine serum, with medium changes every 2 days. Chondrocytes were maintained as a monolayer in Dulbecco’s modified Eagle’s medium (DMEM) supplemented with 10% fetal bovine serum (FBS) and the appropriate antibiotics. At culture day 10, cells were treated as indicated for each experiment.

### 2.4. Sucrose Gradient for Mitochondria Fraction

Primary chondrocytes were lysed by repeated vortexing in 5X volumes of lysis buffer, after which cellular debris was removed by centrifugation [18,19,20]. Lysate were then loaded onto a 10%–80% sucrose gradient and centrifuged for 6 h at 15,000 RPM. Equal volumes of lysate fractions were separated by polyacrylamide gel electrophoresis, transferred to nitrocellulose membranes, and immunoblotted for mitochondrial protein. 

### 2.5. Behavioral Testing

Pain behavior tests for mechanical allodynia were performed using von Frey filaments, as described previously [21]. Rats and mice were placed on an elevated metal grid, with 50% (over five times out of 10) withdraw threshold values determined using the up-down method.

### 2.6. Antibodies and Reagents

The experiments were performed from the following sources: LC3B (Cell signaling, 2775S), p62 (Sigma-Aldrich, P0067), Pink1 (Novus Biologicals, NBP1-39667), Parkin (Abcam, ab77924), MFN-1 (Santa Cruz, D-10), DRP-1 (BD Bioscience, 611738), mtp110 (Santa Cruz, sc-58807). Mitotracker dyes from Invitrogen were used to detect mitochondria. To determine cell viability, assay reagent (EZ-Cytox) was purchased from Daeillab Service.

### 2.7. Quantitative Real-Time PCR

Total RNA was isolated using TRIzol Reagent (GeneAll, RoboEx^TM^, Thermo Fisher Scientific, Waltham, MA, USA), and cDNA was prepared from total RNA using the Kit (Enzynomics, B201, Daejeon, Korea) according to the manufacturers’ instructions. qPCR was performed using the AriaMax Real-time PCR system (Agilent technologies) with the Top real ^TM^ qPCR 2X premix (SYBR Green with low ROX Supplementary Material). Normal chondrocytes were cultured in 6-well plates, and RNA was extracted with TRIzol Reagent (GeneAll, RoboEx^TM^, Thermo Fisher Scientific, Waltham, MA, USA). Three micrograms of total RNA was reverse-transcribed to first-strand complementary DNA (cDNA). Specificity of the reactions was confirmed by 2% agarose gel electrophoresis.

### 2.8. Statistical Analysis

Statistically significant differences between two groups were determined by Student’s *t*-test. Differences among multiple groups were determined by the two-way analysis of variance (ANOVA) followed by an appropriate multiple comparison test. The results are reported as the mean ± SEM. *p* values < 0.05 were considered significant. All data presented in this study are representative of at least three independent experiments. 

## 3. Results

### 3.1. Pink1 and Parkin Expressions Increase in a MIA-Induced Osteoarthritis Model

**Figure 1:**
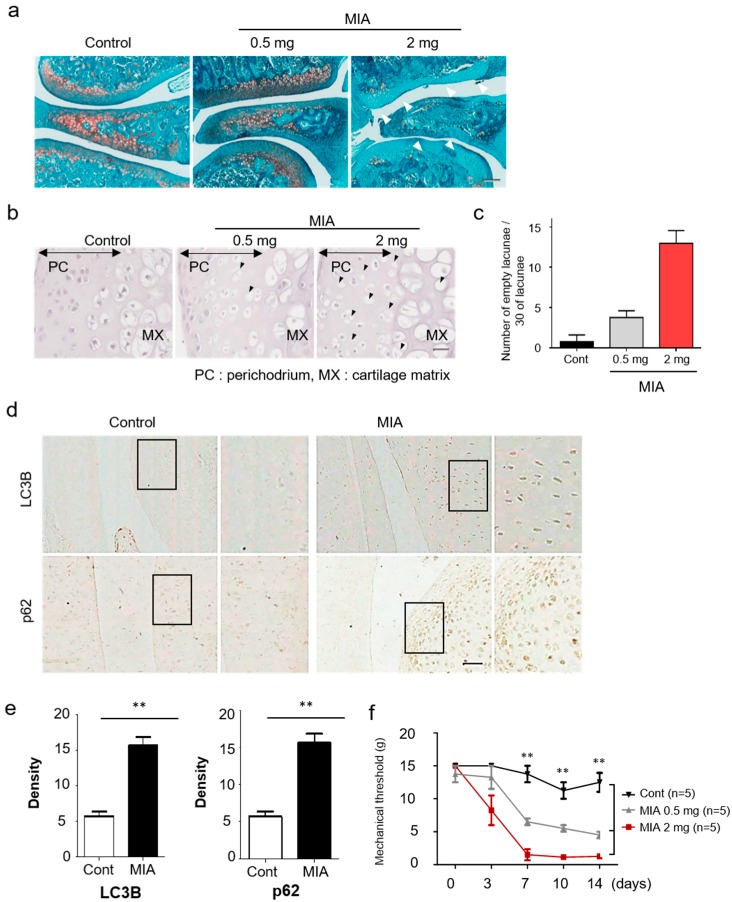
Increased autophagy and cartilage damage in a monosodium iodoacetate (MIA)-induced osteoarthritis model. (**a**) A histologic feature of knee joints in the MIA-induced osteoarthritis (OA) model. At 3 days after injection, the joint sections were stained with Safranin-O/Fast Green. (**b**) Representative hematoxylin stained sections of knee joints on dependent of MIA dose after 3 days. Black arrowheads indicate the chondrocytic cell death in damaged cartilage areas. (**c**) Quantitative analysis for empty lacunae due to chondrocytes death. (**d**) Expression of LC3B and p62 in knee cartilage was measured by immunohistochemistry. (**e**) The density of each protein was quantified with Image J. (**f**) Rats were subjected to behavioral tests using von Frey filaments to evaluate the effect of MIA-induced OA. Scale bar = 50 µm.

As a proof-of-concept, we injected MIA into the knees of rats to assess the level of autophagy induction in a toxin-induced OA animal model. Histology of the resulting knee joints was then examined by Safranin O-Fast Green staining (Figure 1a). MIA-induced significant cartilage damage in the medial tibial plateau and femoral condyle at 3 days post-intra-articular injection.

In the previous work with human primary chondrocytes, MIA was shown to induce apoptosis by producing intracellular reactive oxygen species [22]. To assess the suitability of the MIA-induced OA model, hematoxylin staining of affected joints was used to investigate the cellular and molecular mechanisms involved in MIA-induced chondrocytic cell death. Affected rat joints displayed an altered articular cartilage morphology, including microstructural erosion and matrix loss, as evidenced by hematoxylin staining. Chondrocytes showed a significant increase in the presence of empty lacunae, as compared to the sham group (Figure 1b,c). In addition, chondrocytes in the MIA-induced OA group revealed strong LC3B and p62 immunostaining, which is indicative of autophagy progression (Figure 1d,e). Immunofluorescence staining for LC3B and p62 were chosen due to the strong link between autophagic flux, LC3B, and p62 expression. Mechanical allodynia was observed significantly from approximately 7 days in the MIA-injected group compared to the control (Figure 1f).

**Figure 2:**
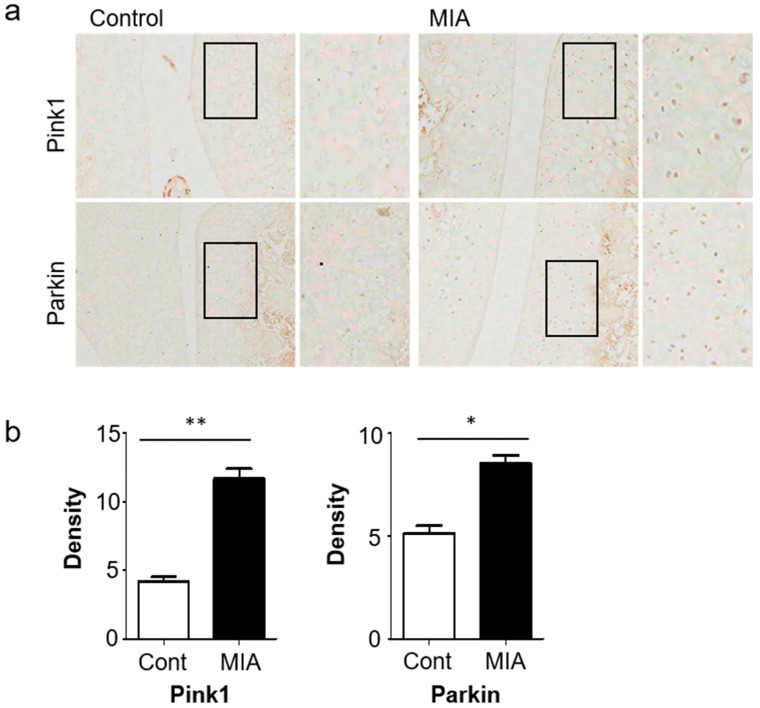
The histopathology of knee joint sections of OA rats demonstrated high levels of mitophagy-related genes. (**a**) Expression levels of Pink1 and Parkin in knee cartilage of OA animal models after 3 days. (**b**) Image J analysis of staining intensity. Scale bar = 50 µm.

Of note, expression of autophagic markers of cartilage degradation were significantly increased at 3 days post-MIA treatment, relative to the control group. Changes in autophagy and mitophagy of chondrocytes during OA progression were verified via histological analyses, revealing strong expression of Pink1 and Parkin in MIA-induced OA rat knees (Figure 2a,b). Together, these results demonstrated increased autophagy and cartilage degeneration in MIA-induced OA rats. 

### 3.2. Pink1-Mediated Mitophagy is Involved in Mitochondrial Fragmentation and Cell Death in Human Primary Chondrocytes

**Figure 3:**
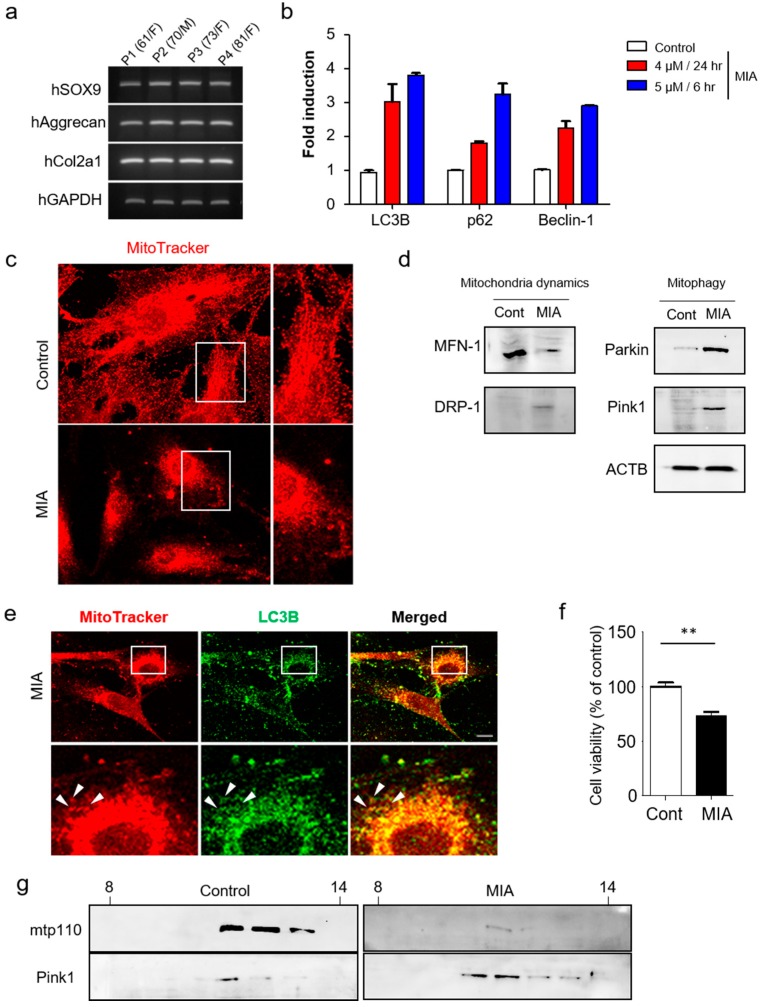
MIA-induced activation of mitophagy in human primary chondrocytes. (**a**) Expression of chondrocyte marker genes (*SOX9*, *Aggrecan*, *Col2a1*) were measured by RT-PCR in human primary chondrocytes. (**b**) Expression of autophagy related genes were detected by qRT-PCR analysis. (**c**) Mitotracker-red and DAPI staining in MIA-treated (5 µM for 6 h) and control chondrocytes. A higher magnification confocal microscopic image from the selected area is also shown. (**d**) Western blot analysis of mitophagy and mitochondrial dynamic proteins in MIA-treated and control chondrocytes. (**e**) Confocal microscopy images displaying subcellular localization of LC3B (green) and mitotracker (red). White arrows indicate encapsulated mitochondria by LC3B. (**f**) Chondrocytes were examined for MIA-induced cytotoxicity (5 µM treatment for 6 h). (**g**) Western blots were used to detect Pink1 and mtp110 levels in sucrose gradient fractions (30%–60%). Scale bars = 50 µm.

To understand more fully the mechanisms by which autophagy limits cartilage damage, we first examined expression of chondrogenic markers *SOX9*, *Aggrecan*, and *Col2a1* in human primary chondrocytes by RT-PCR (Figure 3a). *SOX9*, *Aggrecan*, and *Col2a1* are all cartilage-specific genes [23,24,25], with *Col2a1* serving as a marker for early chondrogenic differentiation, while aggrecan is a major sulfated proteoglycan of the cartilage matrix and a highly specific marker of differentiated chondrocytes. High expression levels of all three genes were evident in chondrogenic cells. Next, chondrocytes were pretreated with MIA for 3 h, resulting in significant, dose-dependent increases in *LC3B*, *p62*, and *Beclin-1* expression (Figure 3b). 

Next, mitotracker-red and DAPI staining were used to confirm the effect of MIA on mitochondrial structure, revealing significant mitochondrial fragmentation in MIA-treated cells (Figure 3c). Subsequent confirmation was performed via staining for mitochondrial fusion and fission-related proteins including MFN-1 and DRP-1. The mammalian homolog of yeast and Drosophila, mitofusin (Mfn) 1, is essential for mitochondria fusion and maintenance of mitochondrial morphology [26], while DRP-1 serves as a core component of the mitochondrial division machinery [27]. Expression of these proteins in MIA-treated and untreated cells indicates that DRP-1 is largely indispensable for mitochondrial fragmentation induced by MIA treatment. While MFN-1 was reduced in MIA treatment compared to control, DRP-1 was consistently induced in response to MIA (Figure 3d). These results further support the notion that DRP-1 functions as a part of the mitochondrial fission machinery in MIA.

Further analysis revealed increased expression of Pink1 and Parkin in the knee cartilage of MIA-treated rats. As mitochondrial fragmentation arises as a result of the imbalance between mitochondrial fission and fusion, we investigated whether defective mitochondrial fusion contributes to Pink1-mediated mitophagy. We first established the direct effect of mitophagy in human chondrocytes after MIA treatment. In addition to exploring mitochondrial autophagy, we performed double-immunofluorescence staining for mitotracker-red and the autophagy-associated protein LC3B. LC3B was shown to co-localize with mitochondria (Figure 3e) at levels high enough that autophagosome accumulation was observed as early as 24 h post-MIA treatment. To observe whether MIA treatment affected cell viability, the cells were incubated for 6 h alone or in the presence of 5 µM MIA. Following treatment, cell viability was decreased to approximately 70% of their pre-treatment levels (Figure 3f), indicating severe mitochondrial damage [28]. To demonstrate more convincingly that the subcellular localization of Pink1 was affected in mitophagy by MIA treatment, Pink1 was detected in mitochondria fractions obtained via sucrose density gradient (Figure 3g). Pink1 accumulates on the mitochondrial surface where it recruits Parkin from the cytosol, which in turn mediates the mitophagic destruction of mitochondria. Further analysis of sucrose density fractions confirmed that MIA-induced activation of mitophagy was associated with Pink1 expression. Taken together, these findings further support the role for Pink1-mediated mitophagy in the regulation of chondrocytic cell death in OA.

### 3.3. Pink1 Knock-Out Decreases Cartilage Damage in MIA-Induced Osteoarthritis

**Figure 4:**
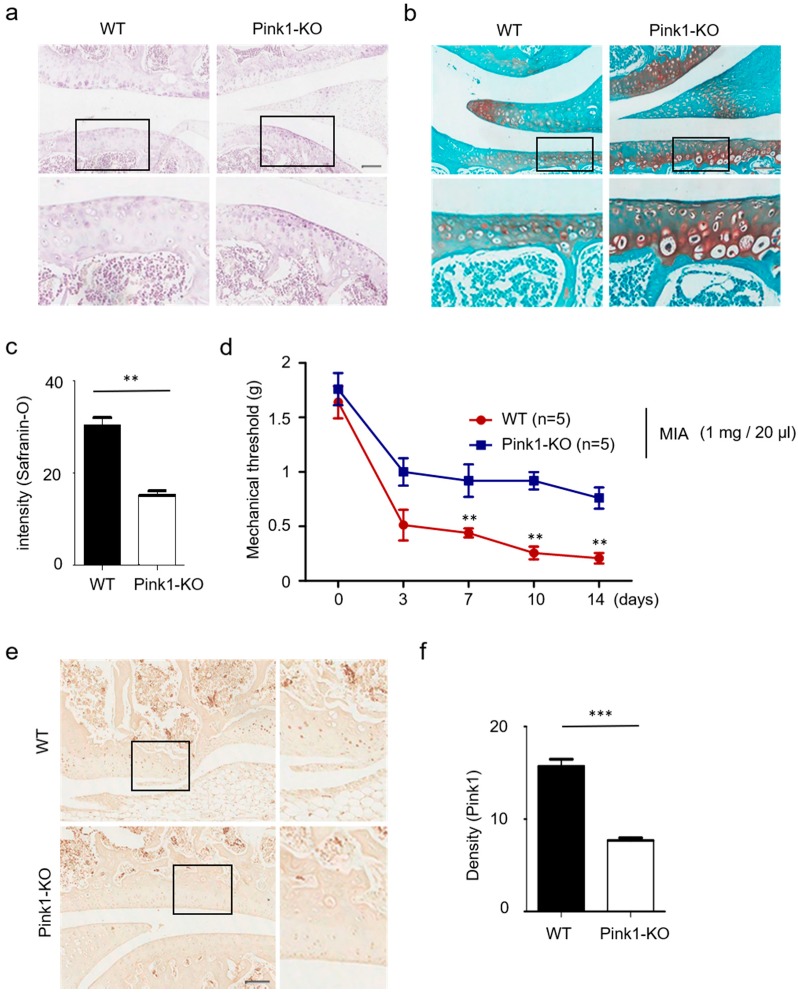
Reduced cartilage damage in Pink1-deficient mice. (**a**,**b**) Hematoxylin and safranin-O staining of knee cartilage treated with 1 mg/20 µL MIA for 3 days. Pink1 knockout decreased knee cartilage destruction in MIA-treated mice. (**c**) Quantification of cartilage destruction and mitophagy-associated autophagic chondrocyte death in safranin-O staining. (**d**) The mechanical threshold was measured on days 0, 3, 7, 10, and 14 after MIA injection by von Frey test. (**e**) Increased Pink1 immuno-reactivity was observed in knee joints of Pink1 knockout mice relative to littermate controls. (**f**) Quantitative analysis of Pink1 expression in mouse Pink1 knockout chondrocytes by Image J. Scale bars = 50 µM.

Using a MIA-induced animal model of OA, we previously showed that overexpression of Pink1 is central to its role in mitophagy. Here, we sought to determine whether suppression of Pink1 expression would be sufficient to prevent cartilage degeneration in MIA-induced OA rats. We first attempted to establish Pink1 deficiency in vivo using a Pink1 knock-out (KO) mouse. As presented in Figure 4a,b, a statistically significant decrease of cartilage degeneration and mitophagy-associated autophagic cell death in chondrocytes was observed in a Pink1 KO mouse (Figure 4c). Based on these observations, we then performed behavioral tests in Pink1 KO mice after MIA injection (Figure 4d). Unlike the littermate control mouse, MIA-induced mechanical allodynia was shown to be significantly decreased in Pink1 KO mice as early as day 3 post-treatment. Expression of Pink1 was also examined via immunostaining (Figure 4e,f). These data demonstrated that Pink1 deficiency alleviates MIA-induced cartilage damage. 

### 3.4. Autophagy and Mitophagy-Related Genes are Highly Expressed in Human Osteoarthritic Cartilage

**Figure 5:**
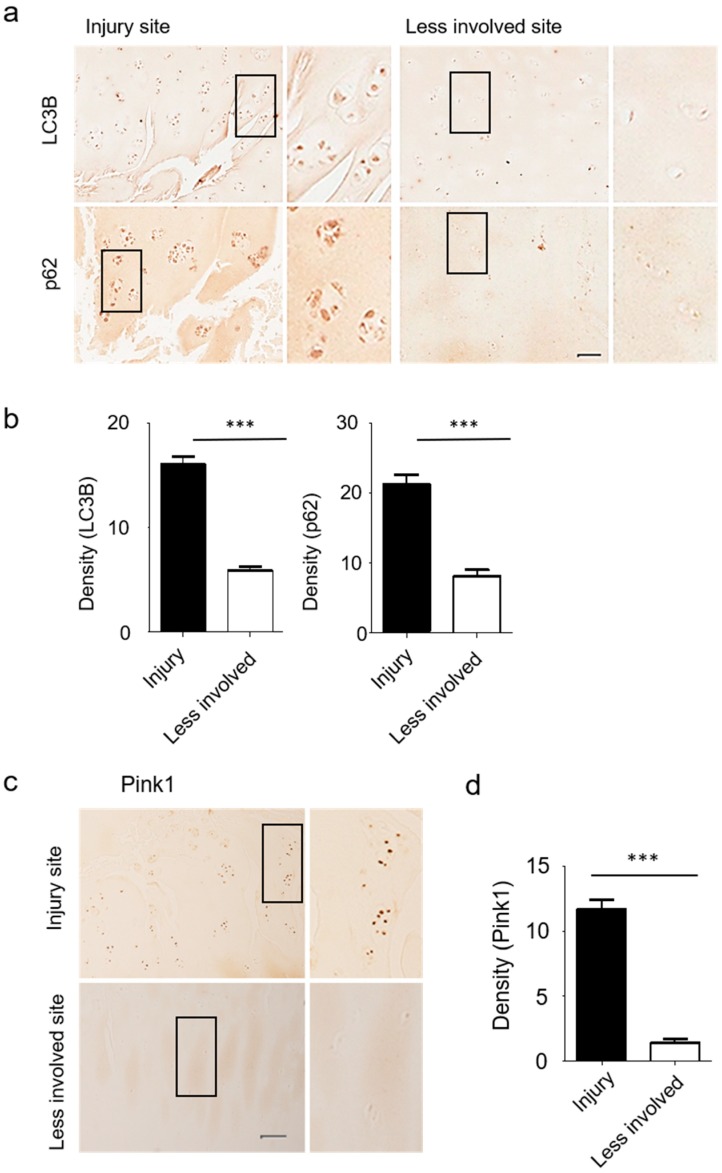
Cartilage from human OA patients revealed increased autophagy and mitophagy-related gene expression levels. (**a**,**b**) Expression of LC3B and p62 were prominent at the site of cartilage injury in OA patients. (**c**) Expression of Pink1 protein in human OA knee cartilage was measured by immunohistochemistry. (**d**) The density of Pink1 in injured and less involved site was quantified with Image J. Scale bar = 50 µm.

Strong induction of LC3B and p62 protein expression was observed in the superficial zone of degenerating human articular cartilage in OA patients (Figure 5a,b). Immunohistochemistry analyses further revealed increases in Pink1-positive chondrocyte numbers in osteoarthritic joint cartilage, with Pink1 expression levels consistently higher in injured sites relative to less involved areas (Figure 5c,d). 

## 4. Discussion

Among patients with degenerative arthritis, there remains a diverse spectrum of inflammatory progression and cartilage destruction, though the extent to which these symptoms are driven by a mitochondria-related genetic predisposition is not known [29]. Animal models of OA typically rely on destabilization of the medial meniscus or resection of anterior cruciate ligament. Despite the fact that chemically induced models may not replicate many of the mechanical factors underlying OA progression, they remain a useful tool for translational research, providing insights into the biochemical mechanism underlying pathology, as well as revealing potential targets for drug therapy. Many studies have shown that the death of chondrocytes is a hallmark of cartilage degeneration in OA [30,31]; however, the mechanisms responsible for chondrocyte death in OA-affected cartilage remain largely unknown. Recently, apoptotic cell death was reported as a dominant event in the degeneration of osteoarthritic cartilage [32].

Recent studies have implicated energy imbalances and decreased activity of several nutrient sensors in human knee OA chondrocytes, relative to their respective normal counterparts [33]. Recent studies have also demonstrated a key role for metabolism in inflammatory joint diseases [34]. In particular, metabolism is drastically altered in osteoarthritis, with aberrant metabolism presenting as a key feature underlying many OA phenotypes. Autophagy plays a crucial role in maintaining cellular metabolism and homeostasis [35]. Autophagy is a self-degradative process that is important for balancing sources of energy at critical times in development and in response to nutrient stress [36].

Mitochondrial mitophagy has been shown to arise as a result of oxidative stress, with previous studies demonstrating a close relationship to chondrocyte damage [37,38]. Genes involved in mitochondrial fusion include Mfn1, Mfn2, and Opa1, while Drp1 and Fis1 are essential for fission. These mitochondrial dynamics have been found to be active under normal conditions but decrease with age and in combination with degenerative changes. Increased mitophagy has been reported in areas of increased fission due to a combination of extracellular stress and the disruption of mitochondrial membrane potential. Therefore, the process of removing damaged mitochondria through fission and mitophagy is a crucial factor in controlling mitochondrial function during cell damage.

In this study, autophagy and cartilage damage were increased in an MIA-induced OA model. An important feature of OA is the abnormal accumulation of damaged proteins and organelles such as mitochondria. Autophagy is an essential homeostatic mechanism, regulating the elimination of damaged macromolecules and protecting functions necessary for cell survival. Some studies have suggested that chondrocytic mitochondria are swollen in OA cartilage. Furthermore, chondrocytes revealed a reduction in the mitochondrial membrane potential [39], consistent with a state of mitochondrial stress. 

Pink1 facilitates the aggregation of depolarized mitochondria in cases of severe mitochondrial damage. Although Pink1 is well-established as a mitochondrial-targeted protein, its expression is highly heterogeneous, having been reported in association with the inner mitochondrial membrane, the mitochondrial inter-membrane space, the outer mitochondrial membrane, and even the cytoplasm [40].

Mitophagy has been known to have protective or detrimental effects on cell survival that is dependent on the level of mitophagy [41,42]. In HT22 hippocampal neuronal cells, glutamate-induced excitotoxicity was associated with mitophagy-mediated mitochondrial activation [42]. In our study, we observed consistent results for Pink1-mediated chondrocytic mitophagy in human primary chondrocytes, MIA-induced OA rats, and Pink1-knockout mice. Pink1-mediated mitophagy is involved in mitochondrial fragmentation and cell death in human primary chondrocytes as well as the rat OA model. Mitophagy-related genes were also highly expressed in human osteoarthritic cartilage. We confirmed these novel phenomena in Pink1 knockout mice, which revealed significant attenuation of cartilage damage in response to MIA challenge. Furthermore, the protective effect of Pink1 deficiency on cartilage damage substantially decreased MIA-treated mechanical allodynia as early as day 3 post-treatment. Moreover, Parkin-independent mitophagy rather than the Pink1-independent one has been suggested in previous studies [43,44], suggesting a feasible clinical application for Pink1-targeted therapeutics.

The data presented here confirm the strong link between mitophagy and osteoarthritis, with Pink1 expression serving as an important regulator of mitophagy-related cartilage degeneration. This study represents the first analysis demonstrating an attenuation of mitophagy and cartilage degeneration in instances of Pink1-deficiency. Further understanding of the pathophysiology linking mitophagy-induced cartilage damage and Pink1 expression may pave the way for targeted osteoarthritis treatments. 

## Supplementary Materials

The following are available online at https://www.mdpi.com/2077-0383/8/11/1849/s1. The primer sequences designed for target genes.
