# Supplementary material for: Pink1-Mediated Chondrocytic Mitophagy Contributes to Cartilage Degeneration in Osteoarthritis
Source: J Clin Med. 2019 Nov 2;8(11):1849. doi: 10.3390/jcm8111849 (PMC6912334; doi:10.3390/jcm8111849)
Supplement: Supplementary file 1 [file jcm-08-01849-s001.pdf]

Journal of
Clinical Medicine

MDPI

Supplementary Material. The primer sequences designed for target genes

The primers used for *LC3B*, *p62*, and *Beclin-1* were as follows: *LC3B* 5′- GGT GAG AAG CAG CTT CCT GT-3′ and antisense 5′- AGA TTG GTG TGG AGA CGC TG -3′, *p62* 5′- GGG CTT TGG TAC CTG GTA CA3′ and antisense 5′- AGG AGG TGG AGG CAA AGG TA-3′, *Beclin-1* 5′-GGA CAC TCA GCT CAA CGT CA-3′ and antisense 5′- TTT CCA CGT CTT CCA GCT CC-3′. Predesigned primers for human *hAggrecan*, *hSOX9*, *hCol2a1*, and *hGAPDH* were as follows: *hAggrecan* 5′-CTACGACGCCATCTGCTACA-3′, and antisense 5′-GGCTTCACCCTCAGTGATGT-3′; *hSOX9* 5′-ATCAAGACGGAGCAGCTGAG-3′, and antisense 5′-GAGCTGGAGTTCTGGTGGTC-3′; *hCol2a1* 5′-GGCTCCCAGAACATCACCTA-3′, and antisense 5′-ATCCTTCAGGGCAGTGTACG-3′; *hGAPDH* 5′-TTCAGCTCTGGGATGACCTT-3′, and antisense 5′- CTCATGACCACAGTCCATGC-3′.
